# Regulated religion, fading belief: how Indonesians’ religiosity has quietly changed, 2000–2020

**DOI:** 10.3389/fsoc.2025.1698036

**Published:** 2026-01-12

**Authors:** Amika Wardana

**Affiliations:** Faculty of Social and Political Sciences, Universitas Negeri Yogyakarta, Yogyakarta, Indonesia

**Keywords:** Indonesian society, religion, religiosity, secularization, World Values Survey

## Abstract

Indonesia is a majority Muslim country with a significant influence of religion in public and private life. Islam, alongside the five others officially recognized religions, serves as a crucial moral foundation within the country’s diverse cultural and governmental frameworks. However, the role of religion has been subject to debate over the years up to the recent politico-religious polarization observed during the last three presidential elections (2014, 2019, and 2024). Drawing from contemporary secularization theory, this study aims to explore the continuity and change in the societal role of religion in Indonesia over the past two decades. It tests the potential decline of religiosity among the populace amidst the continuing strong state’s regulation on religion in the country. The study collects and analyzes secondary data from *Badan Pusat Statistik* (BPS or the Office of Indonesian National Statistics) and the World Values Survey to investigate continuities and shifts in religious adherence and affiliation, levels of socio-religious participation, and individual religiosity—including belief in God and frequency of daily or weekly prayer. The findings indicate that religious adherence and affiliation remain relatively high, coupled with a decent level of socio-religious participation. However, there is a decline in personal religious belief among the population. The contradiction between those three forms of religiosity—affiliation, participation and belief—might suggest an early stage of long-term secularization. In the last two decades, religion is often viewed as a formal identity marker in Indonesian public life, with more nuanced correlation to individuals’ personal religious beliefs and practices.

## Introduction

Religion occupies a central place in Indonesian society, with more than in 10 Indonesians reporting that faith is fundamental to their lives. Few countries illustrate the enduring power of religion in modern public and private life as clearly as Indonesia, the world’s largest Muslim-majority nation. A 2018 PEW research report shows that 93% of Indonesians consider religion very important, placing the country among the most religious nations globally—alongside Pakistan (94%), Ethiopia (98%), Nigeria (88%), and Honduras (90%)—and in sharp contrast to Western Europe, East Asia and Australia, where only 10–18% say the same (PEW Research Center, 2018). From bustling urban centers to remote rural communities, religion continues to saturate Indonesia’s social fabric, anchoring identities and shaping everyday experience even as the country undergoes rapid economic political and technological transformation. The different trajectories of religiosity across the world reflect how modernity and globalization intersect with local histories and social structures (Eisenstadt, 2000; Furseth, 2021). In general, societies with long-standing liberal democratic institutions and high levels of security tend to move toward secularization, while those marked by strong traditional-conservative values and persistent vulnerabilities often sustain or even intensify religious beliefs, practices and authorities (Inglehart, 2021; Norris and Inglehart, 2011, 2015). Indonesia thus represents a compelling case of a society in which religion not only persists but thrives amid rapid modernization and globalization.

The increasing significance of religion in the majority Muslim Indonesian society has been well-documented since the 1990s, when industrialization, modernization, and globalization paved the way for the resurgence of religions, particularly Islam, in the country (Hefner, 1987, 2017; Liddle, 1996). Soeharto’s authoritarian New Order regime suppressed any political activism based on religion but allowed the proliferation of religious organizations and movements with programs and activities in education, health services, social welfare provisions, and religious missions. This resulted in Muslim religious elites and Islamic organizations diverting their energies and resources away from the political field and spreading their influence over the populace by offering various public services alongside traditional religious services (Kim, 2013; Ricklefs, 2012). In combination with the policy of religionization where religion becomes an important and formal identity marker, the state utilized religious doctrines and sources to justify government programs and policies, while also intervening to regulate the religious life of the populace (Menchik, 2016; Ropi, 2017).

Consequently, religion has not only become a prominent feature of the private lives of Indonesians but also an important influence in the public sphere (Hasan, 2009; Zuhri, 2016). Religious beliefs and institutions have continued to play a significant role within modern Indonesian society, shaping various aspects of social, cultural, economic, and political life. Mosques, churches, and temples have become not only religious and cultural landmarks but also centers of activism in both rural and urban areas, which later expanded to the use of digital media to reach wider audiences (Haryanto, 2019).

Yet, the current prominent role of religion in Indonesian society, with special reference to Islam as adhered to by the majority, is not without challenges. As illustrated in the classic socio-political clash between *abangan* vs. *putihan* (non-religious/secularized groups vs. religious-Islamic ones) since the late colonial period and the early post-independence era (Burhani, 2017; Geertz, 1960; Ricklefs, 2007), there have been diverse-cum-contesting societal values dividing Indonesian society. Despite the state ideology of Pancasila accommodating both secular and religious aspirations, the Indonesian public sphere has been a contested arena between the promotion of religious values and the preservation of plurality in terms of religious neutrality and secularity (Hefner, 2018; Marshall, 2018).

The recent return of sectarian politics as presented in the politico-religious polarization in the last three elections (2014, 2019, 2024) (Soderborg and Muhtadi, 2023; Warburton et al., 2020) hints that the persistence of those contestations kept haunting the social (and political) life of Indonesian society. Aside from those facts, the relatively underperformed faith-based (Islamic) political parties compared to secular or non-religious ones in those elections (Fox and Menchik, 2023; Mujani et al., 2018) might narrate a somewhat different story of the quite limited role of religion, particularly in the realm of politics. This raises an intriguing caution that the prominent public visibility of religion particularly Islam should not be taken for granted as not equivalently reflected in shaping and influencing wider social, cultural, economic, and political life of plural Indonesian society.

The study provides a detailed analysis of the continuity and changes in the position of religion in the modern Indonesian society over the past two decades. Despite being considered a religious nation, the influence and role of religion in public and private spheres have not progressed straightforwardly and have been always open to contestation. The increasing significance of religion in modern Indonesian society is not without limits, as it has different effects on society and individuals. It struggles to maintain its importance, faces challenges from its opponents, and may eventually decline. This study is inspired by the ongoing debates on the secularization thesis, which suggests that the outcomes of modernization may vary in different societies, but will certainly impact and change the fate of religion though with diverse forms neither short nor long terms (Bruce, 2011; Bruce and Voas, 2023; Gorski and Altınordu, 2008; Gülalp, 2025). Indonesia, as a majority Muslim nation and considered as highly religious, might have experienced a long-term secularization (Stolz and Voas, 2023) where the intergenerational transmission of religion gradually slipped overtime, weakening belief and attachment amid the strong religious affiliation and institutions. The process has happened along the modernization, the graduate increase of socio-economic prosperities, the preserved political stabilities and particularly the success of mass schooling in the country for the last decades. There has been a slight erosion of religious beliefs and attachments among young Indonesians despite maintain their religious affiliation (Eshtiyagh, 2022; Masuda and Yudhistira, 2020).

This study is a long-term investigation using a descriptive quantitative approach to examine the continuity and changes in religion and religiosity within the broader social context of Indonesian society from 2000 to 2020. The period represents the country’s prominent historical development, with rapid democratization offering more open yet contested public spheres for various religious and non-religious actors. The continuing strong state’s regulation on religion, inherited from the previous authoritarian regime, has kept haunting the social life of the society and transformed into the political arena along with the rise of Islamic populism and political-religious polarization in recent years. The study aims to identify continuity and shifts in the societal role of religion amidst those social changes within this majority Muslim society. It addresses religious affiliations of the populace, participation in socio-religious organizations and activities and their patterns of religiosity in terms of belief in God, the afterlife, heaven and hell and changes in ritual-spiritual practices. The findings offer a comprehensive understanding of the evolution of religion, changes in religiosity, and their impact on social life amidst ongoing processes of modernization, globalization, and democratization over the past two decades.

To achieve the goals, I analyzed secondary data from the official publications of *Badan Pusat Statistik* (BPS or the Indonesia’s Office of National Statistics). I combined this data with the results of the World Values Survey on Indonesia spanning a period of about 20 years. The BPS data includes census reports from 2000 (Badan Pusat Statistik, 2001), 2010 (Badan Pusat Statistik, 2012), and 2020 (Badan Pusat Statistik, 2023), as well as reports of social and cultural statistics based on the national survey of social and economy from 2003 (Badan Pusat Statistik, 2003), 2006 (Badan Pusat Statistik, 2007), 2009 (Badan Pusat Statistik, 2010), 2012 (Badan Pusat Statistik, 2013), 2015 (Badan Pusat Statistik, 2016), 2018 (Badan Pusat Statistik, 2019), and 2021 (Badan Pusat Statistik, 2022). Next, I gathered and analyzed reports from World Values Surveys 4 (2000) (Inglehart et al., 2014b), 5 (2006) (Inglehart et al., 2014a), and 7 (2020) (Haerpfer et al., 2022) regarding data from Indonesia. The surveys involved 1,000, 1,500, and 3,200 respondents, respectively. It is important to note that Indonesia was not included in World Values Survey 6 of 2014.

Based on the reports, I examined and mapped out the continuity and change of the religious composition and affiliation, as well as the participation of Indonesians in faith-based organizations, associations, and socio-religious activities. It also addresses continuity and changes in religion/religiosity (such as the importance of religion, religious practices, and beliefs in God, the afterlife, heaven, and hell), and confidence in religious institutions.

## An affiliation that holds: the resilient role of religion in Indonesian identity formation

Religion has significantly established itself as a formal identity marker in this majority Muslim Indonesian society over the past two decades. This status as a religious nation is substantiated by empirical data. As shown in Table 1, derived from the national census data of 2000, 2010, and 2020, approximately 99% of Indonesians have consistently identified with one of the six officially recognized religions. Islam continues to constitute the majority, followed by Protestantism, Catholicism, Hinduism, Buddhism, and Confucianism. The membership numbers of all these religions have increased in tandem with population growth (from 201.2 million in 2000 to 237.6 million in 2010, and 271.3 million in 2020). However, there has been a slight decline in the proportion of Muslims (from 88.2 to 87.2 and 86.83%), Hindus (from 1.8 to 1.7 and 1.74%), and Buddhists (from 0.8 to 0.7 and 0.76%) within the total population. In contrast, the proportion of Protestants and Catholics has experienced a modest increase, from 5.9 to 7 and 7.52%, and from 3 to 3.10%, respectively. Meanwhile, the percentage of Confucianists has remained stable over the last two decades since its formal inclusion as an officially recognized religion in 2001.

**Table 1:** Religious composition and affiliation in Indonesia 2000–2022.

Religion	2000		2010		2020	
	*N*	%	*N*	%	*N*	%
Islam	177,528,772	88.2%	207,176,162	87.2%	235,624,719	86.83%
Protestant	11,820,075	5.9%	16,528,513	7.0%	20,405,772	7.52%
Catholic	6,134,902	3.0%	6,907,873	2.9%	8,410,812	3.10%
Hinduism	3,651,939	1.8%	4,012,116	1.7%	4,680,884	1.73%
Buddhism	1,694,682	0.8%	1,703,254	0.7%	2,050,505	0.76%
Confucianism	0	0.0%	117,091	0.0%	721,400	0.03%
Others	411,629	0.2%	299,671	0.1%	105,057	0.04%
Not stated	–	–	139,582	0.1%	–	–
Not questioned	–	–	757,118	0.3%	–	–
Total population	201,241,999		237,641,380		271,349,889	

Sources: BPS reports on Indonesian population results of 2000, 2010, and 2020 Censuses.

The development of Indonesia’s religious composition over the past two decades confirms the long-term impact of the state’s religionization policy (Hefner, 2011; Picard, 2011). Religion has been institutionalized as a formal and important marker of identity for every citizen, as recorded in nearly all official documents (Laksana and Wood, 2019; Ropi, 2017). This policy leaves no option for Indonesians to choose either no religion or a religion outside the six officially recognized ones. The strong state intervention to promote religious identity alongside the traditionally religious society as experienced in the country might have preserved the continuation of religion as a prominent social (-political) entity. The state is not simply ‘religiously neutral’ (Ichwan, 2011) but also actively promoting the adherence of (officially recognized) religions (Marshall, 2018). The form of ‘religious laicity’ (Madinier, 2022) where the state manages and regulates religions has resulted in the enduring commitment among Indonesians to adhere to a (officially recognized) religion as part of their citizenship attribution. The high proportion of Indonesians to formally adhere to religion (with approximately 99%) could be understood as a collateral consequence of the country’s political system. In short, being Indonesian means being religious as their citizenship identity.

Particular attention is given to the category of ‘the others,’ which includes individuals who adhere to religions or religious beliefs outside the six officially recognized ones. The proportion of this group has been relatively small and has steadily decreased over the past two decades (from 0.2 to 0.1% and then to 0.05%). This category includes minority communities practicing indigenous religions (such as *Parmalim*, *Sunda Wiwitan*, and *Kaharingan*), members of new religious movements, and those following spiritual traditions known as *aliran kebatinan/kepercayaan* (such as *Subud*, *Sumarah*, and *Sapta Dharma*). These groups emerged, were founded, and organized as local responses to the arrival and growing dominance of world religions (Islam and Christianity) during the late colonial period (Ricklefs, 2007, 2012). They became the focus of contentious debates by the Indonesian state shortly after independence in the 1960s up to the current time (Aragon, 2021; Nalle, 2021). The 2017 Constitutional Court’s decree resolved the problem by allowing followers of these religions to list their unique religious identities on their national ID cards outside the six officially recognized religions. Yet, the situation seems to have changed little and confirm its losing influence within the society (Hefner, 2011).

## Moderate religious participation in a devout nation: a quiet shift in Indonesian religiosity?

Beyond the political dimensions influencing the continuation of religion in the country, it is pertinent to explore other aspects of social life. As indicated in Table 2, Indonesians have maintained a consistently high level of sociability over the past two decades, with more than 70% engaging in various neighborhood social activities. Participation in religious-related events remains the most prominent, involving approximately 50–60% of the population, compared to *arisan* (a unique Indonesian rotating savings and social gathering) at about 20%, *gotong royong* (forms of mutual cooperation or community self-help projects) at about 35%, sport activities at about 10% and arts (dances, musical bands) at less than 1%. This trend is also reflected in participation in funerals, which are often organized as part of socio-religious customs and traditions. The sociability of Indonesians is notably religious in nature underscores the continuing influence of religion in the social and cultural life of the multi-ethnic and multi-faith Indonesian society (Haryanto, 2019).

**Table 2:** Socio-religious participation among Indonesians 2000–2021.

Domain of social participation	Year to year percentage of participation					
	2003	2009	2012	2015	2018	2021
Religious	56.98%	52.78%	62.04%	60.84%	61.87%	48.32%
Womanhood	11.23%	–	–	–	–	–
Youth	10.12%	–	–	–	–	–
Crafts	–	0.76%	1.69%	1.19%	1.56%	0.79%
Sports	8.64%	8.19%	12.49%	12.73%	14.40%	6.78%
*Arisan*	34.62%	20.46%	20.93%	21.21%	20.30%	13.58%
Neighborhood	21.84%	–	–	–	–	–
Funeral	40.28%	31.99%	50.10%	62.30%	63.87%	58.11%
*Gotong Royong*	–	–	–	41.64%	42.13%	35.03%
Arts/Music	–	1.26%	2.61%	–	–	–
Other social activities	17.06%	26.21%	34.14%	44.61%	46.82%	23.06%
Overall percentage of social participation	–	71.30%	81.32%	85.43%	85.43%	77.42%

Sources: BPS reports on Indonesian social and cultural statistics of the 2003, 2006, 2009, 2013, 2019, and 2021.

However, with only 50–60% Indonesians participating in socio-religious activities of the 99% declaring to adhere to religion, including a slight decline to 48.32% in 2021 probably due to the government restriction during the Covid-19 pandemic, requires a careful explanation. Despite its strong roles, religion is not the sole socio-cultural source for majority Muslim Indonesians’ sociability in their neighborhoods. About 30–40% of the population have preferred to participate in non-religious social-cultural activities. The preference highlights the persistence of polarization based on religious/non-religious orientations within Indonesian society forged since the late colonial time (Hefner, 2018; Ricklefs, 2007; Soderborg and Muhtadi, 2023). The religious polarization is not new in the country but generally avoided and prevented by any means. The Indonesian society has still been haunted by the wider and bloody political conflicts involving religious versus non-religious groups during the 1960s Communist uprising, killing about 500 thousand to 1 million people, and the 1990s overlapped ethno-religious and economic conflicts with 1,000 death tolls. Any changes in the religious landscape will bring on various consequences either expected or not and intended or not thus interrupting the societal harmony of this multireligious and multiethnic nation.

## Still religious, quite belonging, less believing: a new pattern of Indonesians’ religiosity

The strong affiliation with religion among Indonesians, as discussed earlier, has been accompanied by mixed forms and directions of religiosity and religious vitality for over two decades. Referring to Table 3, which is based on three rounds of the World Values Survey from 2000 to 2020, Indonesians have shown a mixed form of religiosity. Despite the steady increase in a self-declared religious person among Indonesians, from 69.5% in 2000 to 75.3% in 2006 and 90.4% in 2020, as well as formal affiliation with mosques/churches and religious organizations/associations, from 37.5 to 47% respectively, their religious beliefs have shown a steady decrease. The public confidence toward religious institutions has also risen considerably though it fluctuated from 73.9% in 2000 to 67.7% in 2006 and 85% in 2020. They have enjoyed more respect and recognition from the society. Yet, the belief in God has experienced a slight decline, from 99.9% in 2000 to 97% in 2020, while the perception of the importance of God in their lives has shown a considerable negative change, from 97.3% in 2000 to 85.2% in 2006 and 85.5% in 2020. A similar negative trend has been observed in other aspects of religiosity, such as belief in the afterlife (from 99.2% in 2000 to 73.5% in 2020), heaven (from 99.9% in 2000 to 86.8% in 2020), and hell (from 99.7% in 2000 to 69.7% in 2020).

**Table 3:** Dynamic of religious beliefs, practices, and social life in Indonesia 2000–2020.

Dimensions religiosity	WVS4 2000	WVS5 2006	WVS7 2020
Religious persons	69.5%	75.3%	90.4%
Important of God	97.3%	85.2%	85.5%
Believe in God	99.9%	–	97.0%
Believe in After life	99.2%	–	73.5%
Believe in Hell	99.7%	–	69.7%
Believe in Heaven	99.9%	–	85.8%
Praying everyday	–	91.7%	83.1%
Attending religious services (more than once in a week)	30.9%	29.7%	21.4%
Attending religious services (once a week)	64.7%	64.5%	62%
Membership of church or religious organization (active)	–	37.5%	47.0%
Confidence to religious institution	73.9%	67.7%	85.0%

Sources: World value survey country reports of wave 4 (2000), wave 5 (2006), and wave 7 (2020).

The level of religious devotion among Indonesians has remained consistently high, especially in terms of daily prayer, although it has slightly decreased from 91.7% in 2006 to 83.1% in 2020. However, the frequency of attending religious services once a week has been relatively modest, at around 60%, for the last two decades. The percentage of people attending services more than once a week has been quite low and has gradually declined from 30.9% in 2000 to 29.7% in 2006 and 21.4% in 2020. The data suggests Indonesians show high individual religious vitality, but societal participation in religious activities is more modest. This is consistent with the socio-religious participation of the populace presented in Table 2, which is based on the national statistics of BPS, where only 50–60% are actively involved in socio-religious activities either in their neighborhoods or wider Indonesian society.

The data shows that Indonesians have maintained a high level of religiosity and religious vitality over the past two decades. This is like trends seen in Latin America, Africa, and Asia (Inglehart, 2021; Norris and Inglehart, 2011). Despite rapid modernization and democratization since the 2000s, religion continues to play a significant role in Indonesian society, especially in individual religiosity. Similar to non-Western societies, Indonesia is experiencing a trend of increased or sustained religious influence, rather than a gradual process of secularization (Furseth, 2021; Parker, 2019). This desecularization is attributed to a combination of political and religious factors, as well as the open and pluralistic nature of religious activities and services in this predominantly Muslim country (Fealy, 2008; Schmidt, 2017). In summary, religion continues to play important roles at both societal and individual levels in Indonesia; a sign of a ‘still religious’ society.

However, it is important to note that only about 60% of Indonesians regularly attend religious services and take part in religious organizations and activities. The gradual decline in their belief in God, the afterlife, hell, and heaven suggest that there are different reasons for this. This indicates a form of religiosity in Indonesia that involves a sense of belonging but with less emphasis on strong beliefs. The term ‘belonging’ refers to membership and participation in religious groups and activities, while ‘believing’ refers to actual beliefs in God and the sacred (Day, 2011, 2020). These elements are crucial in understanding the changing role of religion in society and at the individual level. This concept was first used to describe secularization in Britain as ‘believing without belonging’ and has since been expanded to Western countries (Davie, 1990, 2012; Tromp et al., 2020). For Indonesians, it is ‘believing’ which is declining amidst the relatively high affiliation and steady ‘belonging’ to religion. The reverse pattern shows a rather different form and process of secularization in non-Western countries, which is culturally religious and with the strong state regulation on religious life.

## Is there a long-term secularization happening in Indonesian society?

Secularization has not fully occurred in developing countries in Asia, Africa, and Latin America due to limited existential security in terms of physical, financial, and wellbeing (Inglehart, 2021; Norris and Inglehart, 2011). However, the influence of Western liberal and secular values, stemming from colonialism and globalization, has impacted social, educational, economic, and political systems in these regions, particularly in Asia including Southeast Asian countries like Indonesia (Six, 2017, 2020). The pattern of Asian secularization, though not mutually exclusive, has been developed along with the creation of modern nation-states, as an effort to keep the position of religion in the society while controlling its influence in the government (Dean and van der Meer, 2019; van der Meer, 2022). It may not follow the traditional and organic secularization identified with declining of the role of religion and religiosity altogether within the society (Bruce, 2011; Gorski and Altınordu, 2008).

The pattern of ‘still religious, quite belonging, less believing’ exhibited by Indonesians suggests the more nuanced and complex socio-cultural, political, and historical processes of secularization, as well as its inevitability affecting most people around the world due to modernization and industrialization. The state policy of religionization (Picard, 2011; Ropi, 2017) or a form of ‘religious laicity’ (Madinier, 2022) to promote religious adherence and regulate religious life, might have unintended consequences by decreasing religious vitality and engagement despite strong personal religious affiliation. The prominent role of *Pancasila*, once known as the way of life, the sole state principle, and societal moral source of the country, resembles Bellah’s idea of (American) civil/public religion, including its secularizing features (Intan, 2023; Intan, 2006). The socio-political forces have encouraged adherence to religion as a legal and formal identity marker for the public life of the populace, but with a modest level of commitments to socio-religious engagements and not fully correlated with religious beliefs in their private life.

Furthermore, this pattern illustrates a different narrative of the resurgence of Islam and religion in the country since the 1990s (Hefner, 2017; Kim, 2013; Liddle, 1996). This is part of the deprivatization (Casanova, 1994) and desecularization of religion around the modern world (Berger, 1999). The high level of religious affiliation, supported by pro-religion state policies, indicates the ongoing and increasing public role and influence of religion. Yet, this might not solely bring in positive impacts for the society, like supporting democratization, human rights and eradicating corruption and so forth (Hefner, 2019), but just utilized by religious actors for their own purposes either for maintain their survivals, social, political or economic benefits (Brown and Fauzia, 2019). It has become a reality check for the resurgence of religion in Indonesia society as it has contributed to the current decline of democracy with promoting illiberal values (Menchik, 2016), populism (Hadiz, 2018; Yilmaz et al., 2022) and recent socio-political polarization (Soderborg and Muhtadi, 2023).

The patterns of ‘still religious, quite belonging, and less believing’ indicate that the Indonesian religious revival since the 1980s–1990s modernization and further the 2000s democratization has reached its limit, declining as socio-political stability and economic prosperity have increased. It might follow a long-term secularization, as religion finally lost its relevance in modern society (Stolz and Voas, 2023). For Indonesians, after decades of religion’s prominent role, they are beginning to show a somewhat different projection. The decline of ‘believe’ is just the beginning, as they see religion as a socio-political and economic capital for personal or communal success, which could be embraced without truly believing in it. The pattern underscores a transformed socio-political and economic meaning of religion, which, in the long run, might erode its relevance within the culturally religious and persisting state supports and religious regulation of Indonesian society. However, this has happened gradually over the years.

## Conclusion

The article shows that the changing pattern of Indonesia’s religious life from 2000 to 2020 might represent a form of long-term secularization. The country had experienced a resurgence of religion, thanks to strong state regulation supporting (official) religions in the 1980s and 1990s and to the broader opportunities during the 2000s’ democratization. As a result, there has been a very high level of religious affiliation over the last two decades. However, it is limited to a legal and formal identity marker for the public life of the populace, but with a modest level of commitment to socio-religious engagements and now declining religious beliefs. The change coincided with the growing negative image of religion as merely social, political and economic capital. The pattern of ‘still religious, quite belonging, less believing’ suggests that the Indonesian religious revival has reached its limit and is now declining amid increased socio-political stability and economic prosperity.

Finally, the study is based on secondary data from the Indonesian office of the National Statistics and the World Values Survey country reports covering the 2000–2020 period. It is not allowed to run sophisticated statistical analysis. Considering the limitations, it calls for further study by tracking cohort dynamics, regional and class variation, and the long-run effects of policy on beliefs and practices across several spheres of life within Indonesian society. It will also call for comparative research in Southeast Asia—such as Malaysia, Thailand, and the Philippines—as their religious landscapes might experience similar dynamics and changes.
